# Evaluation Study on the Use of Non-Contact Prevention and Protection Products in the Context of COVID-19: A Comprehensive Evaluation Method from AHP and Entropy Weight Method

**DOI:** 10.3390/ijerph192416857

**Published:** 2022-12-15

**Authors:** Yanlong Guo, Xuan Li, Denghang Chen, Han Zhang

**Affiliations:** 1 Social Innovation Design Research Centre, Department of Design, Anhui University, Hefei 203106, China; 2 Anhui Institute of Contemporary Studies, Anhui Academy of Social Sciences, Hefei 203106, China; 3 Department of Science and Technology Communication, University of Science and Technology of China, Hefei 203106, China; 4 College of Environmental Science and Engineering, Ocean University of China, Qingdao 266000, China

**Keywords:** contactless, epidemic-proofing products, hierarchical analysis, entropy power method

## Abstract

In the post-epidemic era, there is an endless supply of epidemic prevention products that cover a wide range of public areas. The introduction of such products has eased the tense pattern of virus proliferation in the context of the epidemic, and effectively demonstrated the initiatives implemented by the Chinese people in response to the outbreak. This paper therefore begins with the study of contactless epidemic prevention products, which appear in a form that meets the needs of contemporary society and offers a new mode of living to it. It enriches the measures for epidemic prevention and control. By obtaining satisfaction ratings from the user community, the performance of such products can be understood in time to provide a substantial basis for the subsequent upgrading and optimization or transformation of such products. This study uses the KJ method and questionnaires to construct an index system for contactless epidemic prevention products, grasp users’ needs for epidemic prevention products in real time, classify and identify such products, and select such products as epidemic prevention smart security gates, medical delivery robots, infrared handheld thermometers, thermographic body temperature screening, contactless inductive lift buttons, and contactless medical vending machines. The questionnaire was designed with four dimensions: safety, intelligence, aesthetics and economy. A sample size of 262 was collected through the distribution of questionnaires. We used AHP and entropy weighting methods for the comprehensive evaluation; AHP basically tells us how satisfied most users are with this type of product. The use of the entropy weighting method can achieve objectivity in the weighting process. Combining the two approaches helps to improve the scientific nature of the weighting of the evaluation indexes for contactless and epidemic-proof products. It is clear from the AHP analysis that, firstly, there are differences in the perceptions of the performance of this type of product between different age groups. Secondly, the user group rated the perceived performance of the product presented as high ($B_{n}>0.200$), which users can subjectively and directly perceive. Next, the perceived future sustainable economic development of this product category is low ($B_{n}\leq 0.200$), and users place low importance on its economic aspects as an objective additional condition. The entropy method of analysis shows that, under reasonable government control of the market for intelligent products, the safety, intelligence and aesthetic effects of these products are significant ($C_{m}\leq 0.100$); further, the economic presentation of these products has yet to be optimized and upgraded ($C_{m}>0.100$).

## 1. Introduction

Since the end of 2019, during the novel coronavirus (COVID-19) pandemic, “social distance measures” have been considered the most effective method of outbreak prevention [1,2]. Considering the epidemic situation, traditional self-isolation can no longer stop the spread of the virus [3]. Governments must take the necessary measures to mitigate the spread of the virus [4,5]. In 2020, with the gradual integration of various epidemic prevention products into major public places, the government was able to effectively monitor the spread of the epidemic at a relatively low cost [6], and the epidemic has been effectively contained. It has become a trend for major companies to develop improved designs for epidemic-proof products [7]. 

As we know from the article “Big Data in the Public Health System”, intelligent big data analysis is now being incorporated into all epidemic prevention products in the marketplace. The integration, fragmentation, reconfiguration, mining and research of big data has led to the formation of epidemic prevention products with different functions. Therefore, the use of big data as a carrier support is an inevitable trend [8]. As was the case in past epidemics and pandemic outbreaks, intelligent epidemic prevention products can facilitate the diagnosis of COVID-19 cases [9]. An example is the medical imaging platform. The platform incorporates the state of human lungs into a specific recognition system for the rapid diagnosis of COVID-19 cases, and has promoted the spread of digital technology in the healthcare industry. This has helped in designing products with intelligent functions, such as real-time monitoring, timely tracking and rapid detection, which greatly facilitate patients’ lives and give them a certain level of health protection [10]. At the same time, some designers have used product development and market research to further enhance product performance in the design of this type of health QR code. In addition to features such as ease of loss and perishability, COVID-19 prevalence factors such as viral infection coefficients and epidemic prevention efforts are fully integrated into this type of product [11]. Other influential smart product designs include the Smart Assistant, which addresses both virus elimination and the reduction of personal travel frequency through autonomous action; it is a functional assistance machine that combines intelligent culling, following, and storage [12]. Therefore, from an epidemic health perspective, the demand for epidemic prevention products, especially those based on intelligence, has become the social norm [13].

The research on epidemic prevention products is divided into two main categories, namely, the study of indicators for contact prevention products and the evaluation of indicators for contactless epidemic prevention products. Contact epidemic prevention products include disposable epidemic prevention masks, which have become a daily necessity for consumers in the post-epidemic era [14]. The consumption of this product is huge, placing great pressure on the environment, and it is necessary to promote the green consumption of disposable epidemic prevention masks [15]. Contactless epidemic prevention products are based on contact epidemic prevention products, and a number of contactless epidemic prevention products have been developed and designed for use, many of which combine the painful and difficult points of current epidemic prevention and control, adding a technological line of defense. The introduction of such contactless products is a sustainable form of consumption for the development of society, an ongoing process that needs to be adapted and developed over time [16].

For this reason, a large number of contactless products have been incorporated into the public protection area to combat the spread of the virus. For example, in crowded areas, intelligent 6G systems are used to assist drones and area chains to monitor the mass density and wide movement of people [17]. A contactless inductive lift button has been developed for public lifts where there is no air circulation. Its performance relies on interactive sensing between the user community and the product [18]. Using the principles of reflective projection and stereoscopic imaging, smart technology is incorporated into the application scenario of public lifts [19]. This type of sensor less touch interaction is different from the physical buttons of conventional devices, and is able to completely avoid the risk of cross-infection from physical contact with bacteria and viruses [20]. In public toilets, a contactless solution with the automatic sensor dispensing of a smart hand sanitizer is proposed. This category ensures that body temperature is measured while disinfecting, greatly reducing human and material resources and enabling convenient zero-touch disinfection monitoring [21,22]. A new thermal imaging system and 5G thermal imaging body temperature detection technology are used in metro and high-speed railway stations. The use of non-contact, high-precision, multi-target and non-sensitive passage is in strong contrast to traditional manual monitoring, solving the disadvantages of requiring contact, easy contamination and difficult global control. It has improved the efficiency and accuracy of field operations and has good application prospects and promotion value [23,24]. Infrared handheld thermometers can be used in all types of public areas. This is a smart handheld device based on a combination of intelligent handheld devices and portable infrared lenses, relying on an intelligent operation and inspection control platform to directly acquire and analyze infrared profiles. It provides intelligent auxiliary judgments for equipment overheating conditions, effectively circumvents the tedious post-processing and uploading of reports, and is very easy to use and carry [25]. For this reason, a number of contactless epidemic prevention products for public places have been made available with epidemic-prevention functions. The products are effective in providing timely feedback for people with abnormal body temperatures, effectively containing the spread of the novel coronavirus [26].

A similarly large number of contactless epidemic prevention products have been incorporated into the medical protection area. Medical delivery robots, for example, can plan paths in real time. Such products replace healthcare workers in their daily distribution tasks [27] and are a much-needed technological innovation worldwide today, playing a crucial role in the control of novel coronaviruses [28]. The new form of the drug vending machine (NPM) combines temperature and pulse detection with medicine procurement, breaking the traditional manual retail model and enabling safe and efficient medicine procurement 24 h a day [29]. Automatic medicine vending machines are contactless automated intelligent online pharmacies that can be easily accessible to countless users anytime and anywhere. The intelligent machine can be further optimized still, and in the future, it will be transformed via the incorporation of a doctor using programming techniques to use it to its full potential [30]. These contact-free products are extremely convenient for both medical staff and patients, efficiently circumventing cumbersome contact processes.

The above presentation of contactless vaccination products only offers a study of the designs of individual vaccination products, while a quantitative analysis of contactless vaccination products and an evaluation of the overall factors from a systemic perspective are still missing. This might include the subjective evaluation of users who have used contactless epidemic prevention products to evaluate contactless epidemic prevention products in terms of their intuitive usability. Therefore, this paper makes full use of the relevant research results and some of the design elements to develop an index system for the comprehensive use of contactless epidemic prevention products in the form of a questionnaire. Specifically, the hierarchical analysis method and the entropy weight method are used to evaluate user satisfaction with contactless epidemic prevention products, and on the basis of these evaluation results, an optimization strategy is proposed.

## 2. Overview of Contactless Vaccination Products

In response to the instability and complexity of the new coronavirus (COVID-19) and the increasing demand for epidemic prevention products, various types of epidemic prevention products have been made available on the market at all levels, with the introduction of contactless epidemic prevention products being particularly important for the development of society. The forms in which these appear meet the needs of contemporary society and offer a new mode of living to it. This has enriched the measures that can be taken to prevent and control epidemics.

Contactless epidemic prevention products are based on contact products, and are optimized by adding scientific knowledge. In terms of protection functions, they can be divided into isolation screening materials (such as epidemic prevention intelligent security gates, thermal imaging body temperature screening, etc.), environmental purification materials (such as automatic sensor hand sanitizer, contactless sensor lift buttons, etc.) and convenient prevention materials (such as contactless medical vending machines, medical delivery robots, etc.). They use artificial intelligence, AR and big data analysis as a vehicle of support. The high degree of precision and speed mean these approaches can effectively replace traditional manual ones. The processes involved are simple and easy to understand; the results are good and can be applied to all age groups, among many other advantages [31]. The advanced intelligence of these products can help governments to optimize the spread of epidemics, leading to the spread of digitally intelligent epidemic prevention products, as shown in Table 1.

## 3. Information and Methods

### 3.1. Research Methods

Based on the mass production model of contactless vaccination products, it is clear that a comprehensive evaluation by users is particularly important for this type of product. In order to comprehensively evaluate the contactless vaccination systems, there is a need for user research into their performance attributes, so that they can ensure improved user satisfaction in all aspects. For user research, both quantitative data and qualitative data studies are used. The quantitative information search focused on importing the main indicators and data from the questionnaire into SPSSAU (https://spssau.com/index.html) and applying the data to the entropy method through data analysis. Qualitative information studies are carried out according to hierarchical analysis and the entropy method [32], which is a multidimensional approach to decision making [33]. The entropy weighting method allows for objectivity in the weighting process. The systematic and modeled analysis of the 262 valid data samples from the questionnaire can effectively avoid the subjectivity of demand shifts when analyzing decisions on complex issues. A comprehensive evaluation study was completed using a combination of hierarchical analysis and entropy weighting measures to calculate the ownership importance elements and rank them in order of importance.

### 3.2. Evaluation Indicator System Construction

In conjunction with the Chinese government’s linkage to the current system of epidemic prevention and control products, and with reference to the relevant epidemic prevention and control systems, AHP and entropy methods are used to develop the scientific and reasonable evaluation of contactless epidemic prevention products in four dimensions and with ten indicator systems. The general requirements for the construction of indicators for the evaluation of the use of contactless epidemic prevention products are as follows.

The safety of contactless epidemic prevention products was taken as a starting point. The market for contactless epidemic prevention products is oriented towards the general public, and directly related to the spread of the epidemic and the safety of users; their security is particularly important. The security of the products is given more consideration in the design, starting with the analysis of the product’s lifecycle in terms of the system objectives and the characteristics of the user’s needs [34]. In the field of transportation, for example, an integrated systems analysis framework for the functional and cyber security of automotive products has been proposed [35]. The safety of products for everyday usage has been studied [36]. The natural inclination of man has been followed in the design of the product to enhance its security and practical value. Therefore, contactless prevention products should be evaluated for safety at the beginning of development. Security is the starting point for the product and must be ensured as part of a smooth operation. When constructing the index system, we should consider the product’s own performance and its scope of use to form the B1 safety metric [37], the C1 performance maturity and the C2 operational stability [38,39].

The intelligence of contactless vaccination products is a core issue. With strong government support, artificial intelligence is being commercialized on all fronts. Large industries are gradually replacing manual labor with machines. Contactless vaccination products are being designed to use artificial intelligence and cloud computing to improve overall market competitiveness and user satisfaction during the product development phase. This form of intelligence helps in obtaining results [40]. Most internet companies are now able to track user preferences through effective intelligent products to predict user satisfaction with the product [41,42]. Products are increasingly being combined with big data artificial intelligence, and the business of intelligence is gradually expanding and has taken its place in the consumer market. The performance of face recognition, voice broadcasting, and big data usage and tracking is gradually improving. Focusing on user needs and the timely extraction of valid data for feedback offers a different experience to users and has become prevalent in the market. This pattern of intelligibility, on the other hand, takes a lot of time and effort in development. Therefore, for contactless epidemic prevention products with intelligence as the core, B2 intelligence, C3 real-time monitoring, C4 effective feedback and C5 ease of use are employed in the construction of the index system.

Aesthetics concerns the subjective visual effects of contactless vaccination products. Aesthetics is an element of interesting value that provides value to the product. The subjective visual effect on a person is an important factor in enhancing product satisfaction [43,44]. For example, many researchers have tried to find characteristics of products that are aesthetically pleasing, proposing a functional relationship between aesthetic preference and complexity [45]. The product types create aesthetic impressions, i.e., the feeling that arises from perception. Symbolic association concerns customers’ responses to the product’s features [46,47]. The relevance and realizability of smart products are steadily being developed, vie the further understanding of the user group from an aesthetic point of view. The need to expand aesthetic interest as a subjective visual effect of the product is also based on the pursuit of functional requirements. The attractiveness of the product plays an important role in its marketability. The aesthetic associations of the product include happy, excited, thrilled, bored, depressed, etc. Therefore, adding aesthetic elements to the design of a contactless anti-epidemic product can create a higher product market potential and user satisfaction [48,49]. Imbuing products with clean, simple and pure colors can produce consistent aesthetic associations across age groups as a means of achieving a high subjective visual effect. In constructing the index system, B3 aestheticism, C6 color richness and C7 simplicity of shape were incorporated.

We take the economics of contact-free vaccination products as an objective condition. Products currently on the market are moving towards the circular economy concept. However, the approach to the design of circular products is different from traditional new product development approaches [50]. In the context of today’s ecological consciousness, the design of most products should consider the concept of sustainable design. Therefore, more costs and efforts need to be invested in the research and development of such products. In contrast, contactless epidemic prevention products are based on the concept of sustainability, where the life of the product and the selection of durable materials should be considered first and foremost, allowing for the long-term use of the product. The second consideration is to choose lightweight materials, aimed at facilitating the transport of the product. We must then consider the extent to which the product can be reused, as a high recycling rate of valuable recyclable products will affect the overall design process. Designers should adopt certain design approaches to ensure that this product is easy to transport and protects people from the effects of the epidemic. In this sense, the economy category is taken as an objectively important consideration, as a way of promoting the development of society as a whole. In constructing the indicator system, B4 economy, C8 resource utilization, C9 ease of transport and C10 degree of reuse are considered.

Based on this, a system of guidelines for the evaluation of non-contact epidemic-proof products can be constructed in a general form using four aspects: safety, intelligence, aesthetics and economy. Each of these four aspects can be evaluated via the quality of their specific contents. A system of indicators for evaluating satisfaction with the use of contactless epidemic prevention products is shown in Table 2.

### 3.3. Questionnaire Design

Firstly, 10 attributes were selected to evaluate the use of contactless epidemic prevention products in this questionnaire, which was edited in Questionnaire Star and conducted in an open-ended format. For the open-ended questionnaire, the target population was the whole population, as all people were affected by the epidemic in one way or another; the questionnaire sought to assess changes in individual lifestyle-related behaviors [54]. The programmed strata of Table 1 were transformed into question descriptions and then evaluated. The questionnaire was employed in the form of a scale [55], with each question consisting of a narrative sentence and nine different levels of response for each, and with the scores categorized as 1, 2, 3, 4, 5, 6, 7, 8 and 9 in the order of the degree of effect evaluation, with each score representing the respondent’s evaluation of this set of statements (Table 3). The questionnaire included basic information (gender, age, and education level) and an analysis of satisfaction with the use of contactless vaccination products (e.g., how mature you think the contactless vaccination products are in terms of performance). The questionnaires were accurately distributed in the field and online by research team members with specialist knowledge, at locations such as Anhui University, Hefei University of Technology and the Lotus Community in Hefei. 

## 4. Statistics and Analysis

A total of 286 copies of this questionnaire were distributed, 262 of which were valid, with an efficiency rate of 91.6%. In total, 24 questionnaires were judged to be invalid as some of them were answered in less than 5 s, and all the answers were the same. During the questionnaire period, the user group maintained a positive attitude towards the questionnaire research (Table 4). The questionnaire was imported using SPSSAU and the data were studied using hierarchical analysis and the entropy method, respectively. Among the valid questionnaires, the proportion of men and women was 46.95% and 53.05%, respectively, with the highest proportion of age being 19-30 years old (60.31%) and the lowest proportion being 51 years old and above (8.78%). The highest percentage of academic qualifications was undergraduate at 52.29%, followed by postgraduate at 30.92%. AHP hierarchical and frequency analyses were then conducted on the 262 samples’ data. The weight values of each indicator were derived, and a consistency test was completed.

### 4.1. Confidence and Validity Analysis

A reliability study is a comprehensive analysis based on the validity of the data performed using KMO, commonality, variance, the factor loading coefficient and other indicators to test whether the data are worth using.

The questionnaire data were imported into the SPSSAU statistical software for factor analysis and reliability testing. From the above table, it can be seen that the alpha value of Cronbach is 0.784 (Table 5), which is greater than 0.7, thus indicating the high reliability of the research data; after analysis, the sample data test statistic KMO was 0.803, with a significance level of *p* < 0.05, passing the validity test and satisfying the applicability conditions of factor analysis (Table 6).

Cronbach’s alpha values of 0.70 and above indicate the good reliability of the data. KMO values between 0.8 and 1.0 indicate adequate sampling, while values between 0.7 and 0.8 are still acceptable. 

### 4.2. Weight Calculation and Consistency Test

The audience for the contactless prevention products is the general public, and to ensure consistency in the ranking of the elements, users were randomly selected to participate in the evaluation of the indicators. The nine-level scale of the AHP method was used for the evaluation, and users’ preferences for each element were compared and assigned a quantitative value.

The comparison and assignment of project elements in the product hierarchy model is based on the evaluation scale in the AHP. In order to process the data using mathematical methods, they need to be transformed into a matrix for the purpose of quantifying the findings and determining the importance of the design elements. Let there be m item elements of the contactless vaccination products B1, B2, B3 and Bm; we perform pairwise comparisons between the item elements and transform them into a judgment matrix:

(1) $$B=\left(\begin{array}{c} \;1\;\ldots \;b_{1i}\;\ldots \;b_{1j}\;\ldots \;b_{1m}\;\\ \;b_{i1}\;\ldots \;1\;\ldots \;b_{ij}\;\ldots \;b_{im}\;\\ \;b_{j1}\;\ldots \;b_{ji}\;\ldots \;1\;\ldots \;b_{jm}\\ \;b_{m1}\;\ldots \;b_{mi}\;\ldots \;b_{mj}\;\ldots \;1\; \end{array}\right)={\left(b_{ij}\right)}_{n*n}$$

According to the Perron–Fresenius theorem, the matrix B has a unique non-zero eigen root, and the maximum eigen root $\left(\lambda_{max}\right)$ corresponds to the eigenvector (W):

(2) $$B_{W}=\lambda_{max}W$$

The quasi-measured layer data were subjected to tests as follows:

(3) $$W_{1}\frac{\sqrt{M_{1}}}{\sum_{i}\sqrt{M_{i}}}=0.502,W_{2}=0.497,W_{3}=0.334,W_{4}=0.333\ldots$$

The maximum characteristic root is

(4) $$\lambda_{max}=\frac{1}{n}\overset{n}{\underset{i=1}{\sum }}\frac{{\left(BW\right)}_{i}}{W_{i}}$$

where ${\left(BW\right)}_{i}$ denotes the I component of the vector $B_{W}$.

Based on (1)–(4), the weight values for the quasi-measurement and programmed layer targets can be calculated as

(5) $$CI=\frac{\lambda_{max}-m}{m-1}$$

(6) $$CR=\frac{CI}{RI}$$

where CI is the consistency index, CR is the consistency ratio, and RI is the average random consistency index. 

According to (5)–(6), the consistency index of this matrix is $I=\frac{\lambda_{max}-4}{4-1}=0$, and a query of the average random consistency table shows that RI= 1.490; then, $CR=\frac{CI}{RI}=0<0.1$, so this judgment matrix passed the consistency test. Similarly, the CR values of the remaining judgement matrices were all 0, indicating that all judgement matrices passed the consistency test.

To summarize the above data, a judgement matrix and weights have been calculated for the evaluation of the use of contactless vaccination products, and the results are shown in Table 7, Table 8, Table 9, Table 10, Table 11 and Table 12.

As the table shows, CR<0.1, and this passes the consistency test. From this, the programmed level elements can be weighted and calculated to obtain the comprehensive weight value of the programmed level elements’ objectives—see Table 12.

### 4.3. Determination of Index System Weights Based on Entropy Weight Method

The entropy weight method is used to calculate the entropy weight of the indicator by applying the information entropy after the standardization of the original data, where the formula for standardizing the value $E_{ij}$ when the indicator is positive is:

(7) $$E_{ij}=\frac{F_{ij}-F_{i\;min}}{F_{imax}-F_{i\;min}}$$

The formula for standardizing the value when the indicator is inverse is:

(8) $$E_{ij}=\frac{F_{imax}-F_{ij}}{F_{imax}-F_{i\;min}}$$

where $E_{ij}$ is the original data; i = 1, 2, 3,…, m; j = 1, 2, 3,…, n; i and j denote the needs of the evaluated unit and the number of evaluation indicators, respectively; $F_{imax}$ and $F_{i\;min}$ are the maximum and minimum values of the indicators, respectively.

To calculate the entropy weight of the indicator, the general formula is:

(9) $$E_{j}=-\frac{1}{\ln m}\overset{m}{\underset{i=1}{\sum }}P_{ij}\ln P_{ij}$$

where $E_{j}$ is the entropy weight of the *j*th indicator, m is the number of evaluation indicators, and ln is the natural logarithm function.

According to the calculated information entropy $E_{1}$, $E_{2}$,…, $E_{k}$, the weight $W_{j}$ of each item of information entropy is calculated, and the formula is:

(10) $$W_{j}=\frac{1-E_{j}}{k-\sum_{j=1}^{k}E_{j}}$$

where *k* is the number of impact factors.

As can be seen in the table, the highest weight for C9 is 0.228 and the lowest weight for C2 is 0.045 (Table 13).

### 4.4. Combined Weights Based on the Combination of Hierarchical Analysis and Entropy Weighting Method

Based on the results of the above two methods of assigning weights to the indicators, the combined weight $C_{j}$ is calculated.

(11) $$C_{j}=\frac{\alpha_{i}\beta_{i}}{\sum_{i=1}^{n}\alpha_{i}\beta_{i}}$$

where $w_{i}$ and $W_{j}$ represent the weights of evaluation indexes calculated by hierarchical analysis and the entropy weighting method, respectively. The results of both subjective and objective assignments are synthesized and calculated and are shown in Table 14.

### 4.5. Data Analysis

**Figure 1:**
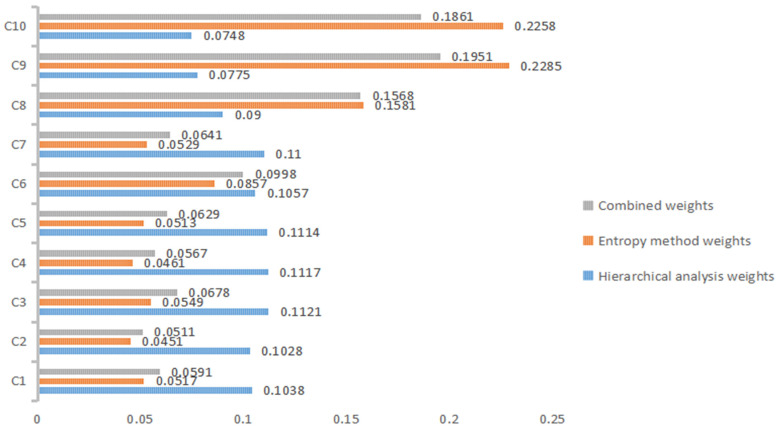
Results of the two assignment methods and combined weights.

After calculation following the recovery of the data, we can obtain the following (as shown in Figure 1): in the hierarchical analysis method—C1 performance maturity and C2 operational stability in B1 safety; C3 real-time monitoring, C4 effective feedback and C5 ease of use in B2 intelligence; C6 color richness and C7 simplicity of shape in B3 aesthetics; C8 resource utilization, C9 ease of transportation and C10 reuse in B4 economy. The weights $w_{i}$ of the degree of reuse are 0.1038, 0.1028, 0.1121, 0.1117, 0.1114, 0.1057, 0.11, 0.09, 0.0775 and 0.0748, respectively. According to the data from the hierarchical analysis, it can be seen that the order of importance of the user evaluation indexes of contactless epidemic prevention products is C3 real-time monitoring, C4 effective feedback, C5 ease of use, C7 simplicity of shape, C6 richness of color, C1 maturity of performance, C2 stability of operation, C8 utilization of resources, C9 ease of transportation, and C10 degree of reuse.

In the entropy weighting method, the weights $W_{j}$ of C1 performance maturity and C2 operational stability in B1 safety, C3 real-time monitoring, C4 effective feedback, and C5 ease of use in B2 intelligence, C6 color richness and C7 styling simplicity in B3 aesthetics, and C8 resource utilization, C9 transportation convenience, and C10 reuse degree in B4 economy, are 0.0517, 0.0451, 0.0549, 0.0461, 0.0513, 0.0857, 0.0529, 0.1581, 0.2285 and 0.2258, respectively. According to the entropy weighting method data, it can be seen that higher entropy values indicate that the data are more confusing, carry less information, have smaller utility values, and thus have smaller weights. Therefore, the order of importance of the user evaluation indexes of contactless epidemic prevention products is C2 operational stability, C4 effective feedback, C5 ease of use, C1 performance maturity, C7 simplicity of shape, C3 real-time monitoring, C6 colorfulness, C8 resource utilization, C9 ease of transportation, and C10 reuse degree.

Among the comprehensive weights, the weights $C_{j}$ for C1 performance maturity and C2 operational stability in B1 safety, C3 real-time monitoring, C4 effective feedback, and C5 ease of use in B2 intelligence, C6 color richness and C7 styling simplicity in B3 aesthetics, and C8 resource utilization, C9 ease of transportation, and C10 reuse degree in B4 economy are all 0.0591, according to the comprehensive weight data, the hierarchical analysis of evaluation weights and the entropy method evaluation of weights. The order of the level of importance level of evaluation indexes of contactless epidemic prevention products is C2 operational stability, C4 effective feedback, C1 performance maturity, C5 ease of use, C7 simplicity of shape, C3 real-time monitoring, C6 colorfulness, C8 resource utilization, C10 reuse degree, and C9 ease of transportation.

## 5. Discussion

The new coronavirus (COVID-19) is different from other common viruses in that it has three main characteristics: long-term destructiveness, extended transmission and mutability. If such catastrophes are not strictly controlled, more irreparable damage will be done to society as a whole [56]. Traditional epidemic prevention products involve human-to-human and human-to-object contact, and thus cannot effectively preclude the spread of the epidemic, meaning the risk of epidemics remains high. Contactless epidemic prevention products are an effective barrier to the spread of epidemics and have been widely adopted and used in epidemic prevention work in a wide range of countries and regions.

Therefore, for developing contactless vaccination products, the Chinese government, the product designer and the user group form a link. This study was carried out to perform a comprehensive evaluation of contactless epidemic prevention products, constructing an evaluation index system questionnaire and quantitatively evaluating contactless epidemic prevention products in terms of both subjective experience and objective analysis.

In the study, the important factors affecting contactless vaccination products are first summarized. Combined with the consideration of the requirements of the whole society in terms of epidemic prevention and control in the post-epidemic era, four quasi-measurement layer evaluation indicators have been formed: B1 safety, B2 intelligence, B3 aesthetics and B4 economy. Two subjective and objective methods of assigning weights have been used to analyze indicator data for contactless vaccination products in conjunction with program-level data [57].

Firstly, intelligibility has the greatest impact on contactless prevention products. The B2 intelligence criterion tier was divided into C3 (real-time monitorability), C4 (effective feedback) and C5 (ease of use), with C3 having the highest weight value. This is due to the fact that real-time monitorability determines whether the category is effective in controlling the spread of the epidemic and allowing it to circulate widely in the market. Therefore, this can be further optimized in the future during product upgrades. For example, the use of such products has spread globally, and there is a lack of confidentiality regarding the privacy of individual users. In the process of future product upgrades, according to the nature of different epidemics, the privacy of individual users needs to be considered in order to respond to the different epidemic prevention needs of a society.

Secondly, aesthetics largely influences contactless and antibiotic products. The aesthetics categories of B3 were C6 (color richness) and C7 (simplicity of shape), with C7 having a higher weighting value. A simple shape is more likely to elicit a high visual rating from users than a richness of color. This is because this type of product is aimed at the general public, and the aesthetic needs of users of all ages are different. Simplicity in the product’s presentation can produce commonalities and satisfy the aesthetic needs of all ages. The product is highly rated in terms of user initiative.

Thirdly, safety affects the use of contactless prevention products. The category of B1 security was divided into C1 (performance maturity) and C2 (operational stability). Among them, the weighting value of C1 is slightly greater than that of C2, which indicates that the user group prefers mature products that can provide a safer user experience. Therefore, this type of product should be developed to promote its safe operation in schools, communities and public places, and it should be popularized for users of different ages in different countries, so that social adoption, user satisfaction and user support can be increased. 

Fourthly, economy has a smaller impact on contactless vaccination products. We divided B4 economy into C8 (degree of resource utilization), C9 (ease of transport) and C10 (degree of reuse) and found that the program-level indicators show a large difference between C8, C9 and C10 compared to the weight values of C1–C7. Economy is an additional condition after the performance of a product has been fully developed, and the economic effect is an important factor to consider when improving resource utilization, ease of transport and degree of reuse. If this effect can be expanded, it will effectively accelerate the green economic transition of epidemic-proof products. Some domestic scholars have developed a theory for the economic evaluation of remanufactured products based on market feasibility, enterprise efficiency and the environment [58]. Therefore, we suggest that market effects need to be considered at the beginning of the market launch of such products, so that they can be made to satisfy users and succeed in the market. With the development of intelligent big data, the subsequent sustainability of the product can be optimized. Enterprise investment is strongly encouraged to achieve a model of small investment and large production, optimizing the transition to a sustainable green economy for future epidemic prevention products.

## 6. Conclusions

Our findings show that, firstly, in the criterion tier B2, smartness has a weighting of 0.276, which is significantly higher than the other indicators, indicating that this is the factor that most user groups care most about when considering contactless prevention products. In the intelligence category, the user group cares more about real-time monitoring. This is because real-time monitoring is the first step in epidemic prevention and control. For example, if a random user enters a public place and the contactless epidemic prevention product is unable to detect the user’s abnormal body temperature in time, it will not be able to enter the information into the feedback process, resulting in the spread of the epidemic in the area and the failure to effectively contain the epidemic. Therefore, real-time monitoring is the most important factor in the overall evaluation of big data.

Secondly, the weighting of B3 aesthetics is 0.267, which has a strong influence on the contactless and epidemic prevention categories. As aesthetics is a valuable element of the product, the color and shape of the product are important factors determining whether it achieves user popularity in the market. In terms of the aesthetic qualities, the user group is more concerned with simplicity of shape. Therefore, we propose to improve the product in terms of visual simplicity in order to reduce the aesthetic over-exertion of user groups of all ages.

Thirdly, the criterion tier of B1 security has a weight of 0.255, which influences the effectiveness of the product. The category of performance maturity here has scored higher compared to operational stability. Therefore, at the beginning of the product launch phase, companies should ensure that the product is mature and operationally stable by testing its performance, which is necessary to ensure this product can start circulating in the market. This is not only the case in China, but also in countries such as the USA, the UK, Australia and Belgium, which place great emphasis on the assessment of the technical maturity of their products.

Fourthly, with a criterion tier weighting of 0.200, the category B4 economy has less of an impact on contactless vaccination products. This is because companies producing this type of product only consider whether they have satisfied the user community after the product has been produced, via whether it has been distributed in the market. It has been found that environmental sustainability has been neglected. Therefore, once the product has been costed, it is important to consider the extent to which it is resource-efficient, easy to transport, and reused and recycled.

Currently, the novel coronavirus (COVID-19) pandemic is still ongoing. If such catastrophes are not strictly controlled, the entire world will suffer more irreparable damage [59]. The introduction of contactless vaccination products has begun to prevent the human–human and human–object contact that occurs with the use of traditional sanitation products. However, due to the large variety and wide distribution of contactless epidemic prevention products, there is a lack of centralization in the attempt to form a complete framework for an epidemic prevention system. The challenge is the emergency deployment of human–material–event resources. The data from this study will help governments and product designers in each country to understand the overall requirements of users of contactless epidemic prevention products, and provide a substantial basis for the subsequent upgrading or transformation of such products. This will lead to a more efficient form of emergency management for major public safety and health events [60].

## Figures and Tables

**Table 1:** Classification of contactless vaccination products.

Product Names	Product Images	Product Functions	Product Features
Anti-epidemic intelligent security gates	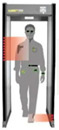	Human security screening Identity capture Personal ID comparison Channel management Video surveillance	High immunity to interference Accurate identification Intelligent remote assistance
Thermographic body temperature screening	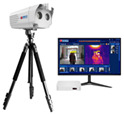	Rapid monitoring of multiple body temperature values Non-contact remote measurement Highly configurable LCD display Accurate automatic temperature correction technology Support for mask recognition	Fast and accurate Safe and concealed Sensitive
Infrared handheld pyrometer	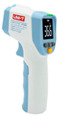	Fast response time Detects surface temperature of objects Wide measuring range Small measurement accuracy with high resolution Temperature measurement of small areas	Easy to carry Durable and drop-resistant Fast sensing
Automatic sensor disinfector	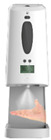	Infrared sensing automatic spraying Adjustable spray angle of spray nozzle Low liquid level warning support Automatic autoclaving	Easy and hygienic to use Intelligent control Wide angle liquid spray
Contactless inductive lift buttons	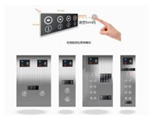	Infrared sensor Voice ride Face recognition Card swipe	Aesthetically pleasing appearance Highly durable materials Precise and reliable sensing Safe and stable performance
Contactless medical vending machines	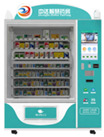	Layering of medicines at different temperatures Environmentally friendly fluorine refrigeration system Explosion-proof tempered glass with multiple anti-theft design Fault diagnosis Smart money payment system	Unmanned retail Fast and convenient Performance and safety
Medical delivery robots	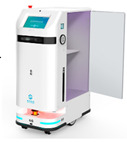	Autonomous door opening and closing Autonomous lift rides Autonomous obstacle avoidance Autonomous charging No human intervention	Highly accurate positioning High transport capacity High efficiency Safe and precise

**Table 2:** AHP-based analysis of satisfaction with the use of contactless vaccination products.

Target Level	Guideline Level	Programmed Level	References
A: Satisfaction analysis of the use of contactless vaccination products based on AHP	B1 Secure	C1 Performance maturity C2 Operational stability	Reimer, S. et al. (2021) [38] Fan, Y. et al. (2022) [39]
	B2 Intelligent	C3 Real-time monitorability C4 Effective feedback C5 Convenience of use	Zhu, L. et al. (2022) [42] Wu, S. et al. (2022) [43]
	B3 Aesthetic	C6 Richness of color C7 Simplicity of styling	Stroncek, D. et al. (2010) [46] Liegeard, J. et al. (2020) [48] Pengnate, S. et al. (2021) [49]
	B4 Economic	C8 Resource utilization C9 Ease of transportation C10 Degree of reuse	Zhou, J. et al. (2021) [51] Zheng, J. et al. (2021) [52] Asriani, D. et al. (2021) [53]

**Table 3:** Description of the indicator conversion questionnaire.

Programmed Level	Description
C1 Performance maturity	What do you think of the performance maturity of contactless vaccination products
C2 Operational stability	How stable do you think the contactless vaccination products are in operation
C3 Real-time monitorability	How well do you think contactless vaccination products are monitored in real time
C4 Effective feedback	How timely do you think the data feedback from contactless vaccination products is
C5 Convenience of use	How fast and convenient do you think the contactless vaccination products are
C6 Richness of color	What do you think of the colorfulness of the contactless vaccination products
C7 Simplicity of styling	What do you think of the simplicity of the styling of the contactless vaccination products
C8 Resource utilization	How resourceful do you think the contactless vaccination products are
C9 Ease of transportation	How easy do you think it is to transport contactless vaccination products
C10 Degree of reuse	How much do you think contactless vaccination products are reused

**Table 4:** Statistical table of questionnaire data.

Title	Options	Frequency	Percentages	Cumulative Percentages
1. Your gender	Male	123	46.95	46.95
	Female	139	53.05	100.00
2. Your age group	18 years and under	26	9.92	9.92
	19–30 years	158	60.31	70.23
	31–50 years	55	20.99	91.22
	Over 51 years	23	8.78	100.00
3. Your highest qualification	Junior Secondary and below	11	4.20	4.20
	Senior High School	38	12.60	16.79
	Undergraduate	137	52.29	69.08
	Postgraduate and above	81	30.92	100.00

**Table 5:** Cronbach’s reliability analysis.

Number of Items	Number of Samples	Cronbach a Cefficient
10	262	0.784

**Table 6:** KMO and Bartlett’s test.

KMO Values		0.803
Bartlett’s test of sphericity	Approximate cardinality	886.400
	df	45
	*p*-value	0.000

**Table 7:** Criterion level judgement matrix and weight values for the evaluation of satisfaction with the use of contact prevention products.

A	B1	B2	B3	B4	$w_{i}$	$\lambda_{max}$	CI	CR
B1	1	0.924	0.958	1.279	0.255	4.000	0.000	0.000
B2	1.082	1	1.036	1.384	0.276			
B3	1.044	0.965	1	1.336	0.267			
B4	0.782	0.723	0.749	1	0.200			

**Table 8:** Judgement matrix and weight values for “security”.

B1	C1	C2	$w_{i}$	$\lambda_{max}$	CI	CR
C1	1	1.009	0.502	2.000	0.000	0.000
C2	0.991	1	0.497			

**Table 9:** Judgement matrix and weighting values for “intelligence”.

B2	C3	C4	C5	$w_{i}$	$\lambda_{max}$	CI	CR
C3	1	1.003	1.006	0.334	3.000	0.000	0.000
C4	0.997	1	1.003	0.333			
C5	0.994	0.997	1	0.332			

**Table 10:** Judgement matrix and weighting values for “aestheticism”.

B3	C6	C7	$w_{i}$	$\lambda_{max}$	CI	CR
C6	1	0.961	0.490	2.000	0.000	0.000
C7	1.041	1	0.509			

**Table 11:** Judgement matrix and weighting values for “economy”.

B4	C8	C9	C10	$w_{i}$	$\lambda_{max}$	CI	CR
C8	1	1.161	1.203	0.333	3.000	0.000	0.000
C9	0.861	1	1.036	0.333			
C10	0.831	0.965	1	0.333			

**Table 12:** Combined weights of evaluation indicators for satisfaction with the use of contactless vaccination products.

Guideline Level	Guideline Layer Weighting Values	Programmed Level	Combined Weighting Values for Programmed Level Elements
B1 Secure	0.255	C1 Performance maturity	0.1038
		C2 Operational stability	0.1028
B2 Intelligent	0.276	C3 Real-time monitorability	0.1121
		C4 Effective feedback	0.1117
		C5 Convenience of use	0.1114
B3 Aesthetic	0.267	C6 Richness of color	0.1057
		C7 Simplicity of styling	0.1100
B4 Economic	0.200	C8 Resource utilization	0.0900
		C9 Ease of transportation	0.0775
		C10 Degree of reuse	0.0748

**Table 13:** Results of the weights of each index determined based on the entropy weight method.

Indicators	Information Entropy Value $E_{j}$	Information Utility Value d	Weighting Factor $W_{j}$
C1	0.9947	0.0053	0.0517
C2	0.9953	0.0047	0.0451
C3	0.9943	0.0057	0.0549
C4	0.9952	0.0048	0.0461
C5	0.9947	0.0053	0.0513
C6	0.9911	0.0089	0.0857
C7	0.9945	0.0055	0.0529
C8	0.9837	0.0163	0.1581
C9	0.9764	0.0236	0.2285
C10	0.9767	0.0233	0.2258

**Table 14:** Results of employing the two assignment methods and combined weights.

Indicators	Hierarchical Analysis Weights $w_{i}$	Entropy Method Weights $W_{j}$	Combined Weights $C_{j}$
C1	0.1038	0.0517	0.0591
C2	0.1028	0.0451	0.0511
C3	0.1121	0.0549	0.0678
C4	0.1117	0.0461	0.0567
C5	0.1114	0.0513	0.0629
C6	0.1057	0.0857	0.0998
C7	0.1100	0.0529	0.0641
C8	0.0900	0.1581	0.1568
C9	0.0775	0.2285	0.1951
C10	0.0748	0.2258	0.1861

## Data Availability

The experiment data used to support the findings of this study are included in the article.
